# Predictive Fault Diagnosis for Ship Photovoltaic Modules Systems Applications

**DOI:** 10.3390/s22062175

**Published:** 2022-03-10

**Authors:** Emilio García, Eduardo Quiles, Ranko Zotovic-Stanisic, Santiago C. Gutiérrez

**Affiliations:** 1 Instituto de Automática e Informática Industrial, Universitat Politècnica de València, Camino de Vera, s/n, 46022 Valencia, Spain; egarciam@isa.upv.es (E.G.); rzotovic@isa.upv.es (R.Z.-S.); 2 Instituto de Diseño y Fabricación (IDF), Universitat Politècnica de València, Camino de Vera, s/n, 46022 Valencia, Spain; scgutier@mcm.upv.es

**Keywords:** marine sensor system, NMEA 2000 network, ship networking technology, PV modules, PV plants, predictive fault diagnosis

## Abstract

In this paper, an application for the management and supervision by predictive fault diagnosis (PFD) of solar power generation systems is developed through a National Marine Electronics Association (NMEA) 2000 smart sensor network. Here, the NMEA 2000 network sensor devices for measuring and supervising the parameters inherent to solar power generation and renewable energy supply are applied. The importance of renewable power generation systems in ships is discussed, as well as the causes of photovoltaic modules (PVMs) aging due to superimposed causes of degradation, which is a natural and inexorable phenomenon that affects photovoltaic installations in a special way. In ships, PVMs are doubly exposed to inclement weather (solar radiation, cold, rain, dust, humidity, snow, wind, electrical storms, etc.), pollution, and a particularly aggressive environment in terms of corrosion. PFD techniques for the real-world installation and safe navigation of PVMs are discussed. A specific method based on the online analysis of the time-series data of random and seasonal I–V parameters is proposed for the comparative trend analyses of solar power generation. The objective is to apply PFD using as predictor symptom parameter (PS) the generated power decrease in affected PVMs. This PFD method allows early fault detection and isolation, whose appearance precedes by an adequate margin of maneuver, from the point of view of maintenance tasks applications. This early detection can stop the cumulative degradation phenomenon that causes the development of the most frequent and dangerous failure modes of solar modules, such as hot-spots. It is concluded that these failure modes can be conveniently diagnosed by performing comparative trend analyses of the measured power parameters by NMEA sensors.

## 1. Introduction

Ships are essentially complex floating systems equipped with navigation, propulsion, power generation, distribution, and other life-support systems. Many controllers, electronic devices, and sensors are part of the equipment on board. Their mission is the permanent supply of information of a diverse nature. Sometimes, this information flow is critical for the safety of the ship, and its loss can place the crew safety at risk. One of the most critical scenarios is the eventual or permanent loss of electricity supply. For this reason, redundant online supervision systems and, especially, predictive fault diagnosis (PFD) systems are of vital importance.

The safety of energy generation and the supervision of its condition, especially of motors, alternators, and inverters, are of a critical nature, where no uncertainty in decision making is acceptable. A PFD is essential for safe navigation based on the permanent analysis of the system’s health status. The objective is to detect and analyze predictive symptoms (PS) that precede system failures obtained from time-series data of system parameters. In this way, they allow operators to have generally adequate margins of reaction in the application of necessary maintenance operations.

In summary, for safe navigation, ships require that many of their systems, especially the power supply and energy storage systems, must have maximum availability [1,2]. If a failure of the power supply and storage systems affects the maneuvering system during a critical operation, the result can be a serious accident [3,4].

In recent years, the use of photovoltaic modules (PVMs) to generate electricity in boats and ships has become widespread. In this way, it is possible to have a facility capable of generating energy autonomously, cleanly and renewably, anywhere. Additionally, PVMs, unlike fuel generators, are completely silent and with less associated cost (fuel consumption).

Nevertheless, PVMs are exposed to various degradation conditions in the particularly aggressive marine environment that affect the constituent materials such as metals, crystals, encapsulating polymers, and especially the photovoltaic cells [5]. These PV cells can experience, due to the special structure of the boats, shading phases on the PVMs that may result in the development of hot-spots. It has been shown that the reduction in power is the best indicator to detect the lack of performance and degradation of a photovoltaic solar installation (PVI) [6].

**Figure 1:**
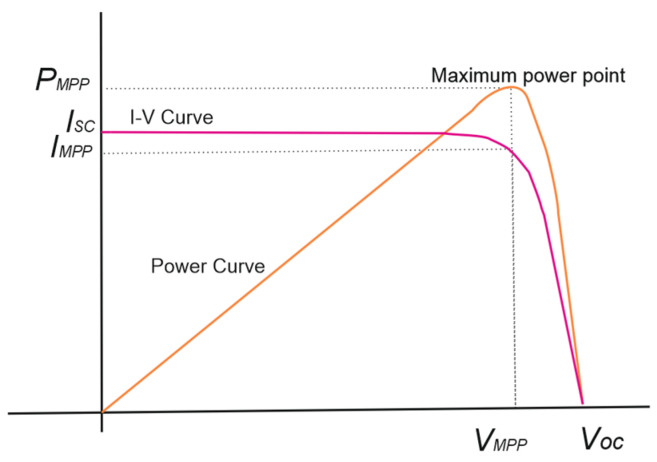
I–V characteristic curve.

PVMs fundamental parameters can be viewed through its “voltage-current” curve, known as the I–V curve. The I–V curve in Figure 1 shows the voltage (*V_MPP_*) and current (*I_MPP_*) parameters at the maximum power point (*P_MPP_*), the open-circuit voltage (*V_OC_*), and the short-circuit current (*I_SC_*) of a module.

Usually, the I–V parameters have not been considered for applying the supervision and diagnosis located in each of the PVMs. It is usually carried out in the inverter connected to each of the strings of the PVMs. This fact has been justified by the argument regarding the increase associated with the cost of the investment to be made, but the recent development of low-cost IoT sensors does not justify this argument nowadays. The PFD techniques focused on each of the panels meet the best conditions to be applied, specifically, using parameters involved in the reduction in the power generated in each of the PVMs, such as I–V parameters. These parameters are strong candidates to be supervised online to apply trend analysis algorithms on the comparative deviation of their dynamic evolution. This supervision allows operators to obtain indicative symptoms prior to reaching a failure condition without recovery possibility.

If the detection of a decrease in power in PVIs is carried out in the inverter, the accumulated decrease in the power produced can be detected, but the possibility of early isolation of the fault centered in the PVM cannot be achieved. This problem can be decisive in knowing on which panel to act immediately regarding its maintenance, in order to disconnect the affected panel, improve the overall performance, and avoid catastrophic failure.

Hot-spot faults stand out for their criticality, frequency of appearance, and critical risk of irreversible damage in PVMs. The appearance of hot-spots in a PVI is a problem of great importance, since it affects not only energy production but also its life span. The appearance of hot-spots produces premature degradation and aging, which is worrying if one considers that the facilities are normally designed to function properly for more than twenty years. Photovoltaic hot-spots have been a well-known phenomenon for more than fifty years [7], one which persists today [8,9]. Frequently the appearance of hot-spots occurs when a panel is partially exposed to shaded areas, and consequently, the affected cell or group of cells operates in reverse polarization conditions, dissipating energy instead of generating it. This fact favors the appearance of hot-spots at very high temperatures, gradually degrading both the generated power and the components of the encapsulation material of the PVM [10,11].

**Figure 2:**
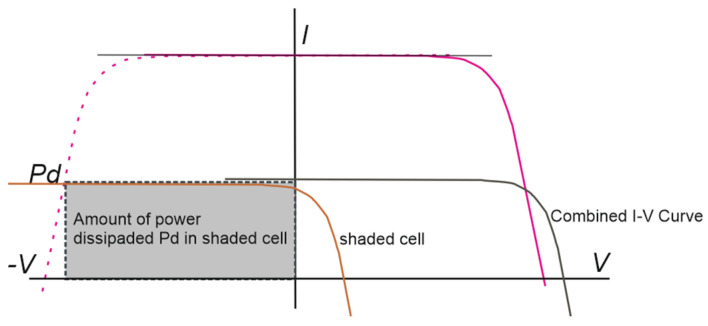
I–V characteristic curve of shading of a cell.

A partial shading on a module, in which a cell of the module is shaded, makes the I–V characteristic curve of that cell vary with respect to the others, since the incident radiation on it is different. This shaded cell can not only not produce electrical current but can consume it by following the power dissipation behavior shown in Figure 2 [12].

When shading occurs on a PVM, depending on the shape of the shadow, the number of cells it covers, and their distribution in the interconnection of the module, the effect of reduction on the generation of electrical energy of the PVM can vary considerably depending on the bypass diodes that it has, and if protection diodes have acted.

**Figure 3:**
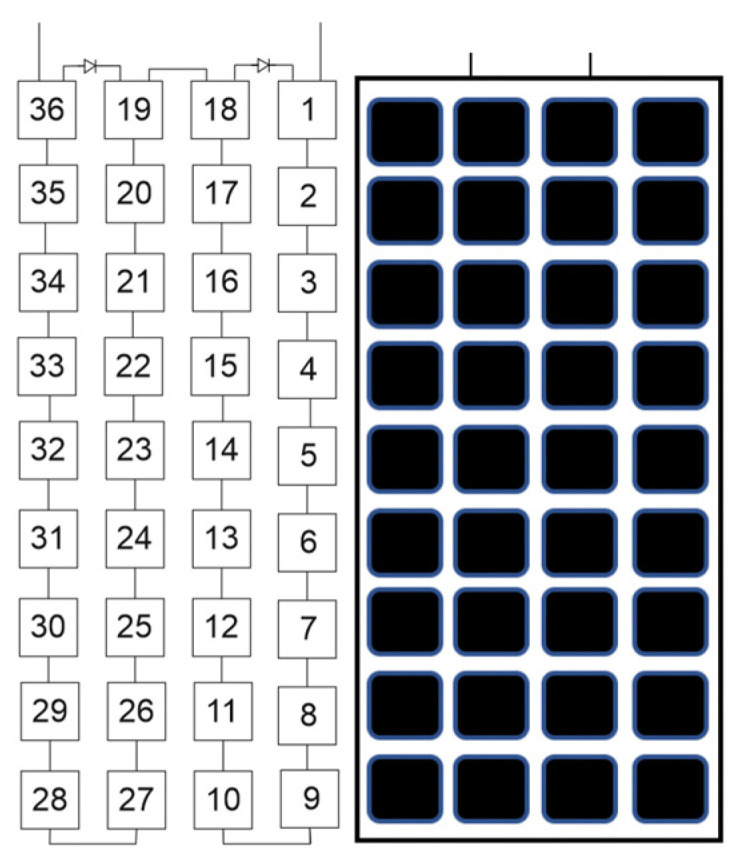
PV panel with two bypass diodes and 36 cells.

Two of the bypass diodes configurations most used by manufacturers, in the type of processes similar to the one used in this study, are with panels of 36 or 60 cells in which (a) 2 diodes are installed, 1 for every 18 cells, and (b) 3 diodes, 1 for every 20 cells, respectively (Figure 3). The decrease in power experienced at the panel in the event of shadowing depends on the number of bypass diodes that are activated in each configuration, oscillating in a decrease in the power between (a) 50% (with two diodes) and (b) 33.3% (with three diodes), if one diode acts in each case to deactivate the cells [13,14]. These energy efficiency losses can be caused by a single fallen leaf from a nearby tree. That is, the possible formation of the hot-spot is avoided, although the decrease in energy efficiency is very considerable.

**Figure 4:**
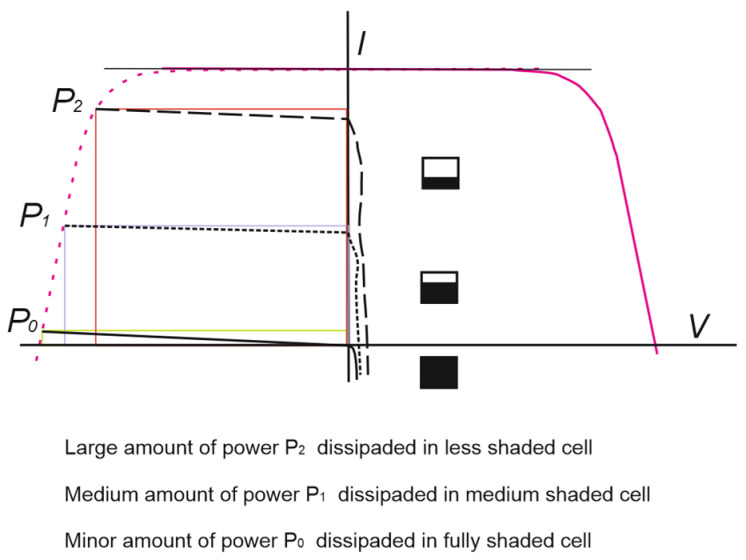
Power dissipation vs. shaded area size.

Figure 4 shows the relationship between the power dissipation of a shaded cell and the size of the shaded area. As can be seen, although the reduction in the shaded area could, in principle, be associated with better operating conditions, the fact that occurs is that the power dissipation, and therefore the degradation phenomenon, increases with respect to that dissipated by a completely shaded cell [15].

In the approach presented in this work, an inductive failure method is applied through different types of gradual shading in one of the PVMs installed in the system.

The motivation of this work is focused on developing a truly PFD method. If the development of an irrecoverable failure is reached, especially a hot-spot development, a destructive and very dangerous fire can take place, especially in open-sea navigation conditions. These situations are not unusual, and the authors have had the opportunity to experience them in person.

One objective is to integrate the PFD algorithm in the context of functional software applications associated with the NMEA 2000 network, especially the N2KExtractor and N2KView, for time series analysis and the management and implementation of the diagnosis method, respectively.

The main contribution of the proposed PFD method is that it is centered and focused on each of the PVMs where the degradation mainly takes place. It works online and in real-time, using observable I–V electrical parameters of easy accessibility. It allows for the incorporation of the possibility of automating actions on the system to avoid future failure event occurrence, detecting not just faults but predictive symptoms.

In Section 2, the NMEA 2000 sensors and devices used in the supervision of photovoltaic electrical energy are presented. The photovoltaic generation system used and the tests carried out are described in detail. The proposed method for onboard PVI supervision and PFD is explained. In Section 3, the results of the experiments are presented. In Section 4, a discussion on results takes place, as well as some additional considerations and findings obtained from the experimental trials. Finally, in Section 5, some conclusions about the advantages of the proposed method for fault identification are drawn.

## 2. Materials and Methods

### 2.1. Materials

#### 2.1.1. NMEA 2000 Smart Sensor Network and Devices

The NMEA 2000 network used to carry out the supervision and diagnosis of the PVI can incorporate photovoltaic parameters through the Victron GX devices. These GX devices have an NMEA 2000 output feature. If enabled, the GX device acts as a bridge: it makes all battery monitors, inverter/chargers, and other products connected to the GX device available on the NMEA 2000 network.

The data is processed with the Vessel Data Recorder VDR100 device of the NMEA 2000 network. This device acts as a black box, acquiring all the data that circulates through the NMEA 2000 network. The acquired data is processed by the N2KExtractor software (Maretron, Carling Technologies Inc., Marine Technology Group, Jupiter, FL 33477, USA) that is installed on a PC connected to the NMEA 2000 network via a USB100 module [16].

The Venus GX device acts as a controller of an energy installation in such a way that a whole series of system components, such as inverters/chargers, solar chargers, and batteries, can be connected to it, working in harmony. Monitoring can be done with the Venus GX locally or from anywhere in the world using an internet connection and the VRM portal. The Venus GX device has an NMEA 2000 output function that justifies its use in this work. In a special way, you have the possibility to connect solar radiation sensors and make their output compatible in an NMEA 2000 network.

**Figure 5:**
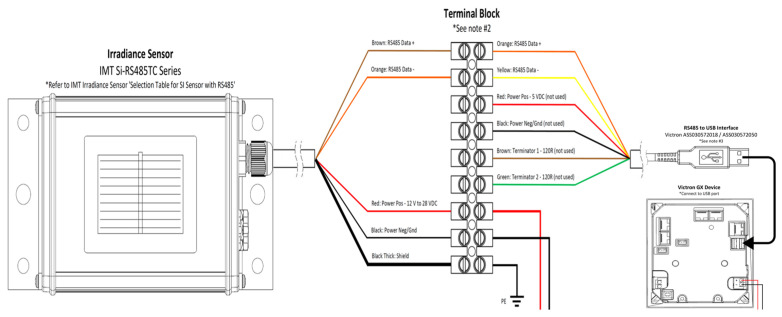
Installation diagram and wiring configuration on a Venus GX controller with Irradiance Sensor IMT Si-RS485TC.

For the measurement of solar irradiance, Silicon irradiance sensors (Si sensors) show a cost-effective and reliable solution. This solution is especially convenient for monitoring photovoltaic (PV) systems due to their spectral response being comparable to PVMs, as well as the similar inclination error (incident angle modifier). It allows for an exact analysis of PV energy yields using Si sensor data [17] (Figure 5).

PB200 weather station has been used in the experimental tests. This instrument outputs time series of weather parameters. PB200 integrates several sensors into a single compact housing. It is equipped with temperature and barometric pressure sensors and sensors for measuring air temperature and true wind speed and direction.

Maretron’s DCM100 module is an advanced electronic sensing device. It can measure DC voltage, current, and temperature of any power source or load. It can be used to monitor PVMs, batteries, alternators, wind generators, inverters, windlasses, or any other DC circuit [16].

To send control actions to the PVMs, the NMEA 2000 network has a DC relay module device such as the DCR100. This model contains 6 direct current (DC) relays, each capable of switching up to 10 Amps. The DCR100 connects directly to an NMEA 2000 network, so it can turn on and off the relays from a Maretron DSM150 or DSM250 display or any PC running Maretron’s N2KView software. The DCR100 easily handles resistive DC loads such as PV modules.

Table 1 summarizes the used devices and sensors, monitored variables and parameters, with their measurement range and accuracy. A more detailed description of the monitoring network can be seen in our previous work in [16].

#### 2.1.2. Experimental PVM Installation

The experimental tests carried out have been applied to a solar plant used for the supplementary energy supply of an ocean-going sailboat with a displacement of 10,000 kg and a length of 14 m, where the concept of safety in navigation is critical. In this type of ship, the occasional shading on the PVMs is relatively frequent, since the architecture of the rigging and the canopy favor it. It has been found that the appearance of hot-spots on the PVMs occurs relatively frequently.

In a solar installation previously installed on the sailboat, it had already been possible to detect the appearance of hot-spots with serious consequences for the energy supply. In 4 years, two of the PVMs had been rendered useless. In the solar installation in another boat with similar characteristics, moored in the same port in 2019, a fire broke out in the PVMs causing considerable damage. This event could have had disastrous consequences, since the fire could have spread to neighboring boats if it had not been because part of the crew happened to be present.

The PVMs chosen to carry out the experimental tests are from the Ecosolar company, with the characteristics shown in Table 2. It is a monocrystalline flexible panel. This option has been chosen mainly because of its weight, adaptability to curved surfaces, and Wp/surface ratio.

**Figure 6:**
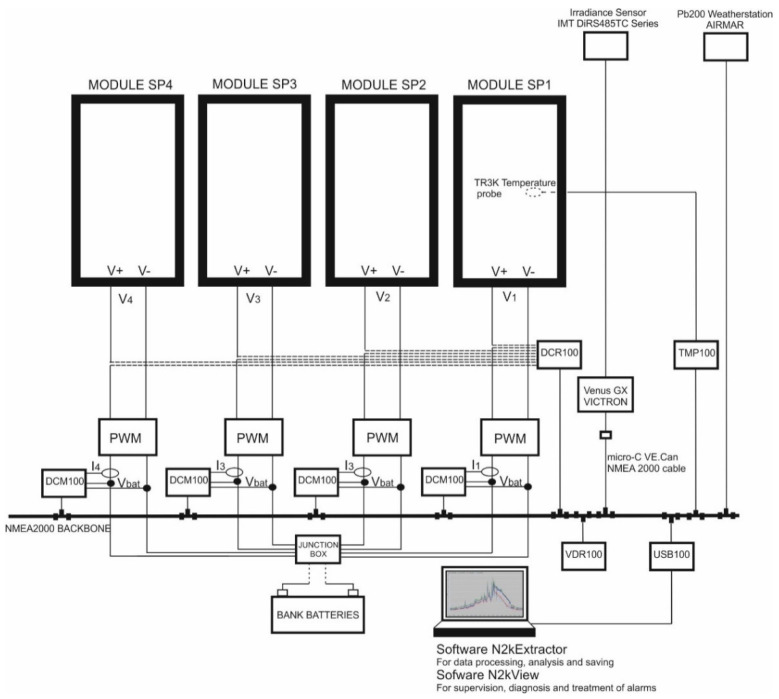
PVMs system installation.

Four PVMs of the type and model described above have been used in this experimental study. The four isolated panels are connected in parallel, each one of them through PWM charge regulators, and connecting the outputs of the latter to a common connection that feeds a system of 12 V service batteries and a total load capacity of 750 Ah. Figure 6 shows the experimental test bench with the data acquisition system and associated sensor devices.

In this type of photovoltaic system, the PVMs are connected in parallel. This is a common characteristic applied in boats, where the power systems and communication networks operate at 12 or 24 volts, not requiring the string connection of PVMs.

**Figure 7:**
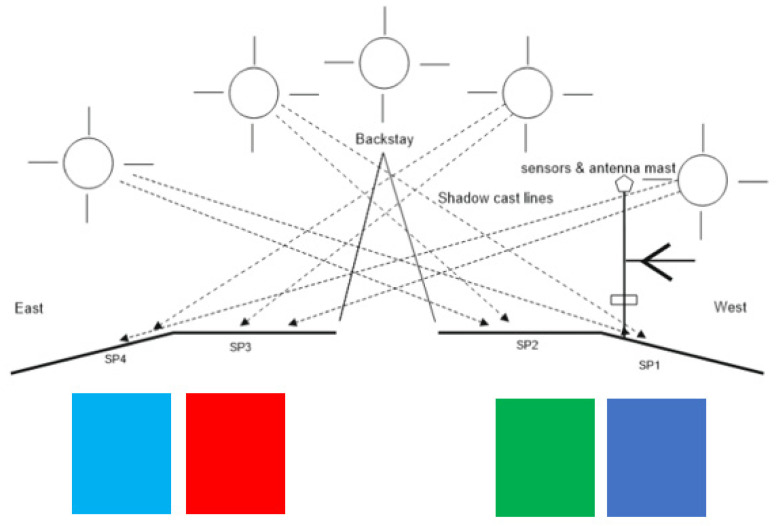
PVMs installation viewed from the bow, with possible projections of shadow lines.

The PVMs were installed in the stern facing south in mooring conditions in port. The SP1 and SP2 PVMs are installed on the port side, and the SP3 and SP4 PVMs are on the starboard side. In Figure 7, the colored squares represent the physical position of each PVM in the installation, showing the color in which they will be represented in the following graphic records of the four colored time series corresponding to the generated currents in each panel.

**Figure 8:**
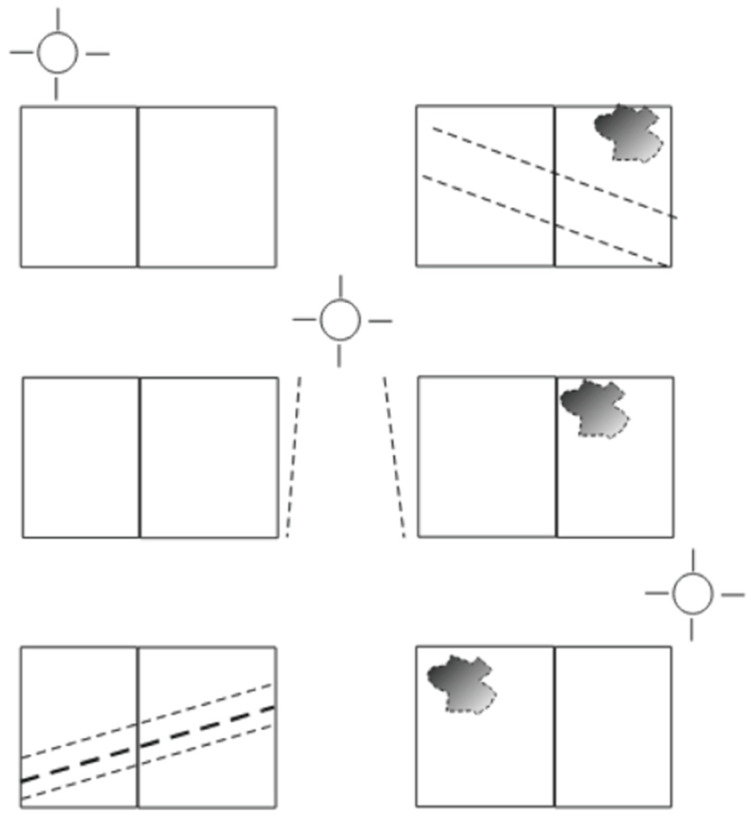
Displacement of the lines and irregular shading areas according to the daily movement of the Sun from east to southwest, in mooring conditions in port.

The spots shown in Figure 8 represent the irregular shadows projected on the panels throughout the day as a function of the position of the Sun. They correspond to the multi-transducer meteorological station and the solar irradiation sensor. The dashed thick line represents the pole where the sensors are located. The thin broken lines represent the projection of the backstay shadow with an inverted-Y shape on the panels.

### 2.2. Fault Diagnosis Method

The online PFD method proposed in this work is based on the execution of constant comparative operations at a certain sampling frequency of the output power generated by the set of PVMs that are part of the PVI. To make the comparison online, it must specifically use convenient parameters, both from the point of view of its accessibility in real-world operating conditions and its symptomatic use correlated with the degradation of the PVMs. This degradation is evidenced through the output power decrease that is generated in each PVM.

As explained in the previous section, the algorithm developed is applied to a small-sized solar installation formed by a set of adjacent and identical PVMs with the same technical characteristics. Due to the reduced assembly space of the PVI, the working hypothesis is to assume that all PVMs will be similarly affected by environmental parameters of temperature, humidity, solar radiation, wind speed, and direction. Consequently, the output power curve that will be generated in the form of a time series with daily and random seasonal components in normal conditions should be very similar, and the result will be adaptive to the superposition of the set of natural disturbances mentioned above. If one of the panels of the set begins to develop in an incipient manner degradation phenomena characteristic of PVMs due to shading, it will do so in the form of a reduction in the power to be generated. An ideal procedure for the early detection and isolation of the fault is to carry out a systematic comparison between the quantitative values of the generated powers and by analyzing the uncorrelated deviations of the time series of the generated power in the PVMs.

However, for practical purposes, it must be considered that the number of electrical parameters to be used to carry out the predictive diagnosis is conditioned by the type of solar regulators used in the installation. If a PWM type regulator is used, which is still widely used in real-world applications, they adapt their operating voltage to that of the solar batteries to which they supply the generated energy, so the voltage values are the same in the input and output of the regulator. Therefore, the variations of the PVM voltage produced by the shading effects are not observable. Consequently, in these conditions, the only observable parameter available to be used is the output current *I_SPi_* at the output of the solar panel regulator.

The PFD algorithm is based on the early detection of uncorrelated point deviations at each sampling event over the data series of the generated output currents *I_SPi_* in each PWM.

The nature of deviations likely to be produced by the effects of the increasing degree of shading correlated with decreasing current deviations can be:

(a) TCD (total current decrease) due to the total activation of the bypass diodes,
(b) CPD (current percent decrease) due to quantized partial activation of bypass diodes, and
(c) MTVPS (minimum threshold value of the predictive symptom) of *I_SPi_* downward deviation for alert declaration observed during the application of the inductive shading method. This type of shading that does not activate the bypass diodes is the one that does not prevent the degradation phenomenon. Consequently, it is the one that introduces the worst conditions regarding the degradation mechanism that remains developing latently.

The behavior of the time series of the quantitative *I_SPi_* values under normal conditions of current generation as a whole shows a great correlation. Partial shading, although reduced in its extension, causes *I_SPi_* reductions up to 1 ampere, producing a significant degrading power dissipation. This MTVPS could be used as a PS.

In this case, the MTVPS was obtained experimentally by applying the inductive shading method. It was first applied on 18 cells of a PVM and, in a second test, on a single solar cell, in periods of 20 min, shading one-third of its surface, two-thirds of its surface and, finally, shading it completely. In both tests, the deviation was 1 A, as can be seen in the Results section.

Therefore, the predictive algorithm for each of the point value samples applies the following search process:

(1) Detect the *I_SPi_* values of the set of measures in the current sample. Consider ${\left(I_{SPi}t\right)}_{t\;=\;1}^{N};\left(I_{SP}t:t\;=\;1,\ldots ,N\right),$ where $I_{SPi}t$ is the series sample observation number *t* (1≤t≤N) for PVM number *i* for *i* ∈ {1,2, …, *k*}, and *N* is the number of sample observations in the entire series (the length of the series). To apply comparative deviation values analysis in the same sampling time *t*, a set with the synchronized sampling *t* series current values of the *k* solar panels (SSCV) such as SSCV = {*I_SP1_t*, *I_SP2_t*, …, *I_SPk_t*} is needed. The *N* observations can be collected in a column vector SSCV = [*I_SP1_t*, *I_SP2_t*, …, *I_SPk_t*]′ of order *N* × 1.
(2) Find the maximum value *I_SPimax_t* from the instantaneous quantitative series values of the current sample in the current vector SSCV. So *I_SPimax_t* ∈ {*I_SP1_t*, *I_SP2_t*, …, *I_SPk_t*}.
(3) The comparative deviations analysis takes place among each one of SSCV elements and the maximum value *MaxI_SPi_t = I_SPimax_t* found in vector SSCV. Calculate the deviations of the other non-maximum series values from the maximum value *I_SPimax_* in the current sample *DevI_SP_t* = *MaxI_SP_t* − *I_SPi_t*.
(4) Find deviations whose condition is *DevI_SP_t* ≥ MTVPS. If the condition *DevI_SPi_t* ≥ *MTVPS* is true, it means that SPi solar panel is suffering a shadowing process.
(5) Activate the alert for SP_i_ disconnection in case of *DevI_SPi_t* ≥ MTVPS as predictor symptom of shadowing occurrence on one or more solar panels.

**Figure 9:**
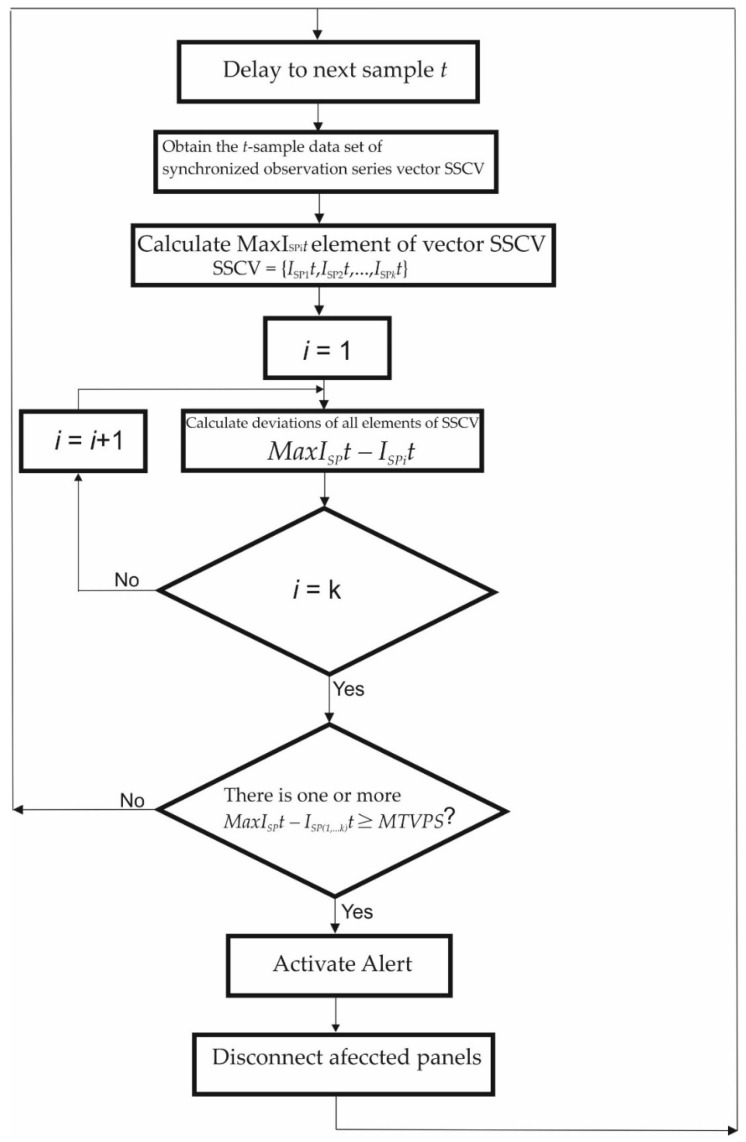
Online deviation trend comparative analysis algorithm, flow chart.

In Figure 9, the deviation trend comparative analysis algorithm flow chart is shown.

## 3. Results

Online monitoring and superimposed recording of the four time-series of the current at the output of the PWM solar regulator is carried out to check the adequacy of the working hypothesis and the comparative method of the deviations produced in the set of time series as a PS of the shadowing event. The data were acquired through the Vessel Data Recorder VDR100. It is a kind of black box that records all types of data circulating through the NMEA 2000 network. It works in solidarity with the N2KExtractor software [16].

Then, the reduction in the current by the induced shading method was used as a PS for the application of the predictive diagnosis in conditions of partial shading of a PVM, establishing the minimum threshold value of the predictive symptom MTVPS, produced by different shading modalities.

**Figure 10:**
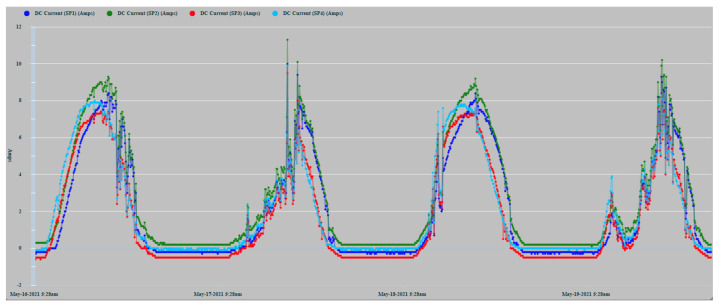
Time series of the current generation of 4 PVMs.

Figure 10 shows the record of the measurements of the current generated by the PVMs SP1 (Navy Blue), SP2 (Green), SP3 (Red), and SP4 (Sky Blue) during days 16, 17, 18, and 19 May 2021. The four time-series of the generated DC current are, in general, highly correlated. However, the correlation is more intense between the two series corresponding to the panels located in the same band. The series of currents generated daily do not present a greater direct correlation because the PVMs that generate them are installed on a curved surface and therefore present differences in their angle of reception of solar irradiation. SP4 slopes to the east, and SP1 slopes to the west.

**Figure 11:**
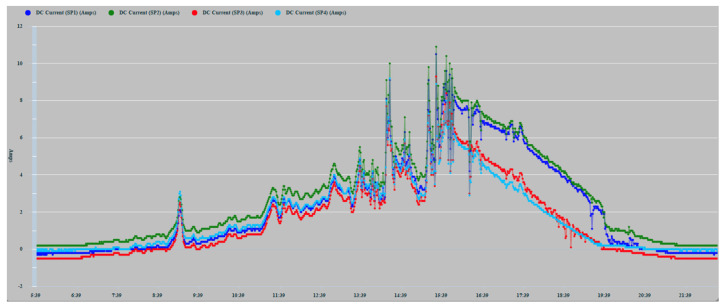
17 May 2021 zoom.

The lack of smoothing of the time series is due to the disturbances caused by the passage of clouds that considerably alter solar irradiation levels. Figure 10 shows how the series corresponding to 16 May 2021, the cloudiness was more intense in the afternoon hours. On the contrary, the series of 17 May showed greater cloudiness in the morning hours. In any case, in the zoom of Figure 11, it can be observed more clearly the random components of higher frequency affected. The four series also show a high correlation because the shading effect was less pronounced in those hours of greater cloudiness. They showed during the afternoon hours with little cloudiness, a greater reduction in the generation of current in SP3 and SP4 because shading occurred with greater intensity.

The two panels, SP2 and SP3, located in the center of the installation (Figure 7), show a better correlation in the hours of greatest solar irradiation because they maintain a flatter and more homogeneous angle of irradiation. On the contrary, PVMs SP1 and SP4 located in each band are tilted, due to their installation in the most pronounced areas of the curvature of the surface. Consequently, they present angles in favor and against the best reception of solar irradiation. SP4 presents a better inclination at dawn and early hours of the day that favors the reception of greater solar irradiation. On the contrary, at dusk, SP1 presents a better angle for better solar irradiation due to its inclination towards the west. As can be seen, this pattern of behavior is repeated in a general way in the four days recorded in Figure 10. It shows that the curves corresponding to the PVMs of the same band maintain a closer correlation with each other, which allows for better observation of possible comparative deviations that may occur due to shading effects.

**Figure 12:**
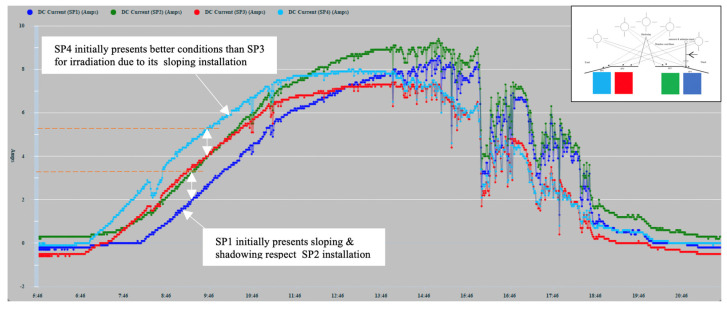
16 May 2021 zoom.

In Figure 12, various types of series deviations are shown, which are justified, both by the effects of shading and by different levels of inclination in the PVMs mounted at the ends of the bands.

**Figure 13:**
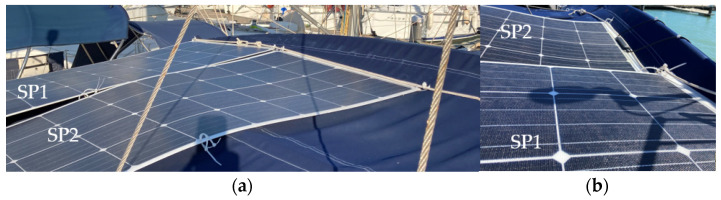
Projection of (**a**) backstay and (**b**) antenna shading lines on SP1 and SP2 in the early hours of the day (9:30 a.m.).

SP4 at the beginning of the day has the best inclination tilted towards the east to receive the solar irradiation in a better condition than SP3, which maintains a flat position. Figure 12 shows a deviation between the two in favor of SP4 that can only be justified because of the difference in inclination between SP4 and SP3, and not because of a possible shading, which was found not to be occurring. In turn, SP3 maintains a flat position identical to the position maintained by SP2. Therefore, there is a high correlation between the two until approximately 10:00 a.m., since it is exposed together with SP1 to shading lines from that time on due to the backstay. During this period, SP1 maintains the most unfavorable position, as it is inclined towards the west, and it is additionally verified that from 10:30 a.m. it begins to experience a combined shading effect that projects two shadow lines due to the location of the backstay (in the form of an inverted Y) of the mast of the sailboat, Figure 13a and additionally the presence of the shadow produced by a mast with antenna and sensors Figure 13b. The projection of shadow lines mentioned above also affects the SP2 module as the Sun advances towards noon (Figure 8); for this reason, SP2 deviates, reducing its generation of current with respect to SP3 that does not experience any shading. In the afternoon, starting at 17:00 h, SP1, due to its more favorable position of inclination towards the west, recovers generation capacity and also because it is no longer shaded as the shadow has shifted towards SP2.

**Figure 14:**
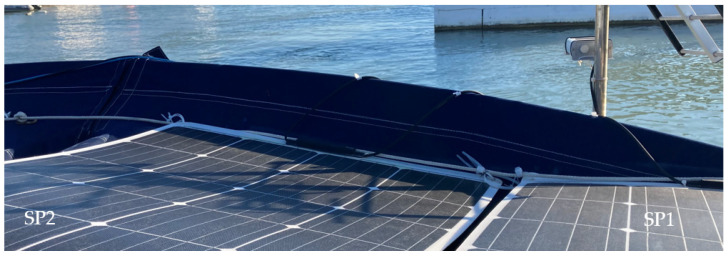
Shading shift from SP1 to SP2 at 16:00 h due to the antenna mast and sensors.

**Figure 15:**
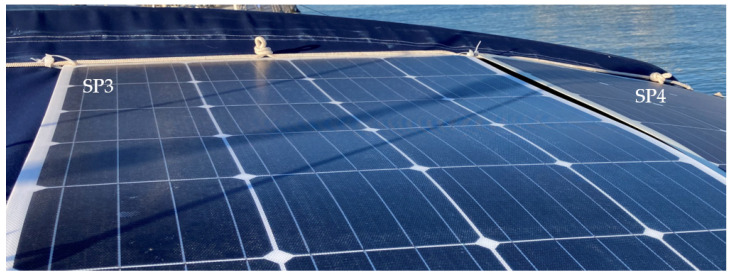
Shaded in SP3 and SP4 at 16:00 h in the position of the Sun at sunset from the Southwest.

Around 16:00 h, the shading of the antenna is moving from SP1 towards SP2 (Figure 14). Additionally, the panels SP3 and SP4 will experience an increase in shading due again to the two lines of the aft backstay and the antenna mast, which move rotating towards the starboard side (Figure 15).

**Figure 16:**
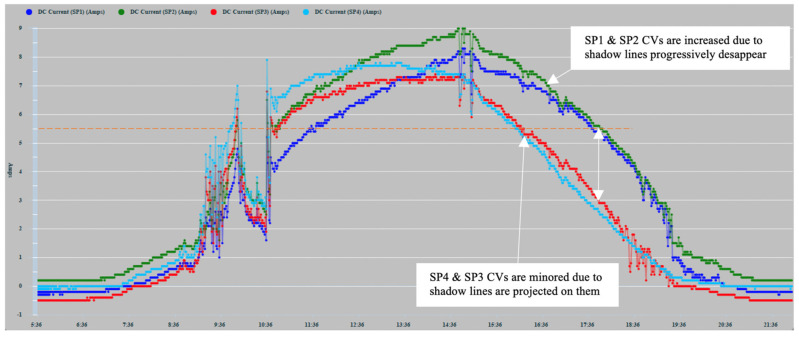
Time series of the generation of current values (CV) of 18 May 2021.

In Figure 16, a detail of the existing correlation between SP1 and SP2 installed on the starboard side is shown. SP1 in its initial evolution presents a reduction with respect to SP2, which is due to the degree of inclination that it has towards the west opposite the sunrise and because from the first hour of the day SP1, it presents a shading effect by the backstays and the mast weather station antenna and solar radiation sensor. From approximately 14:00 h, the deviation gradually lessens due to the shading of the antenna moving from SP1 to SP2, as can be seen in Figure 14.

When estimating the power measurements to be generated, the solar radiation captured must be taken into account considering the existing reception angle since the position of the ship and the degree of inclination also depends on the angle of the heel and the heading of navigation. Through previous measurements made under mooring conditions, the current generation differences of each of the PVMs were favored or impaired, due to the curvature of the installation surface. In the time series corresponding to a panel affected by shading, the deviations due to the decrease in the value of the current will occur in two ways:

(a) Downward deviations that occurred in an abrupt staggered manner in the intensity proportional to the number of affected solar cells activated by the bypass diode;
(b) Deviations of reduction in the generated current due to a partial shading that does not cause activation of the bypass diodes. These cases could be verified by the induction of shading in a single cell and series of cells or by induced shading lines partially traversing a panel. Additionally, random shapes of shadows composed in various ways encompassing more than one solar cell, such as those shown in Figure 14, were formed at certain times of the day. These shadows decreased the current intensity in a range of 1–1.5 (A) in the shadow-induced panel.

The PS to be used among the four current measurements will be detected, observing the possible comparative deviations between the four series.

**Figure 17:**
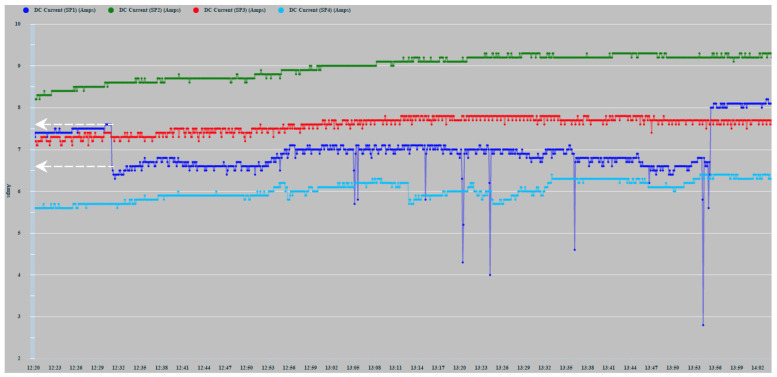
Effect of induced shading on SP1 from 12:30 to 13:55.

In Figure 17, the result of applying the inductive shading method on 6 July 2021, to the SP1 panel is shown from 12:30 p.m. to just before 14:00 h. The method consisted of applying a black plastic sheet completely covering each of the cells belonging to rows 1 and 2 of panel SP1, shading progressively until reaching a total of 18 cells in 5 min intervals. As can be seen, when the first cell was completely covered, a falling flank of approximately 1 Amp was produced (see white arrows in Figure 17). This reduction was maintained with changes in trend throughout the test even though every 5 min, the number of shaded cells increased by one unit. SP1 increased its deviation because additional shadows were cast on SP1 at those times.

**Figure 18:**
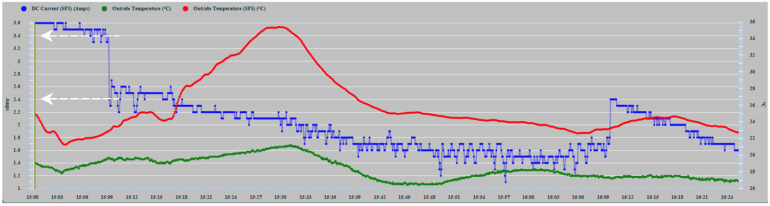
Effect of induced shading on an SP1 cell from 15:10 to 16:20.

Another shading induction test was applied to a single solar cell to quantify the changes that occurred in the power dissipation in the cell as a function of the size of the induced shading area. A temperature sensor was installed at the base of the solar cell on the back of the panel, and the graph of the ambient temperature obtained from the meteorological station was also incorporated for the test. In the new test, a partial shadow was applied to a single solar cell of the PVM with the size of 1/3 of its surface starting at 15:10 h (see white arrows in Figure 18), detecting a falling edge of 1A again. From that moment, a notable increase in temperature was detected from 23.4 °C to 35.3 °C in the 20 min that this phase lasted [15]. In a second phase, from 15:30 h, a shading of 2/3 of its surface was applied in which the temperature of the PVM began to decrease to 24.6 °C. Then, from 15:50 h in a third phase, the shading was applied to the total surface of the solar cell, and finally, at 16:10 h, the temperature dropped to 23.2 °C, at which point all the shading was removed. An increase in temperature can be observed due to the higher level of power dissipation that occurred during the phase of least shading of 1/3 of the cell surface. By causing 2/3 of the shading, the temperature drops notably, which confirms that it is correlated with the decrease in power dissipation, a trend that remains less steep during the total shading phase (Figure 18). The red color corresponds to the temperature obtained by the sensor located on the back of the PVM in the centered position of the solar cell. The green color corresponds to the ambient temperature obtained from the weather station.

The threshold value (TV) has been set experimentally as a predictive symptom of shading from a quantitative deviation of 1A in amplitude. This value has been coincident in the two shading induction tests shown in Figure 17 and Figure 18. In this way, the criterion for obtaining the comparative deviation from the quantitative values of the time series obtained in a set of consecutive samples can be applied. The curve shown in Figure 16 has been used for its practical application, with a sampling frequency of 30 min, for 7 h 30 min.

Table 3 shows the quantitative values obtained by applying the detailed predictive diagnosis algorithm, using the set of time series recorded in Figure 16. In each column, for every sampling, the values obtained from the time series of the PVMs are detailed. The DTV (deviation threshold value) row, in each sample, shows the panels that present a negative comparative deviation greater than 1 A from the maximum value. This deviation is justified by the dynamic evolution of the shading conditioned by the position of the Sun throughout the day.

## 4. Discussion

A search for references related to the diagnosis of faults in solar installations on boats has been carried out to compare the proposed method with similar studies, but little has been found. We have not been able to find anything related to the predictive diagnosis of photovoltaic installations in boats. The references found [18,19,20,21,22,23,24] are basically focused on the study of the weight/PV ratio performance of onboard PVIs.

In the authors’ opinion, as far as we know, no proposal similar to that developed in the work carried out in this article has been produced. This proposal is based on the generation of threshold values of predictive symptoms from the comparative analysis of the deviations that occur in evenly spaced intervals at each sampling instant. The inspiration for the proposed method comes from the knowledge of the operation of the NMEA 2000 network, its characteristics and the requirements of power supplies, which allow for the parallel installation of its PVMs.

Regarding the discriminability of the shadow effect, on sunny days, the shadows originated by the ship’s superstructure are very evident and can be verified if the shadows that appear on the PVMs are correlated with the clearly observable deviations in the graphic records of the time series. In cloudy periods, the series present components of higher frequency, but as a whole, they tend to converge. This means that the shading produced by the clouds is preponderant over that caused by the super-structural elements of the boat, which in cloudy conditions are blurred. In addition, the shading produced by the clouds is tenuous and affects the entire PVM. This fact confirms the initial hypothesis that disturbances affect all PVMs equally. In Figure 18, it can be seen how total shading is less harmful to the degradation of the PVM than partial shading.

Excluding the difference between the degree of orientation between the PVMs, which can be taken into account for their elimination, as can be seen in the graphs, the series under normal conditions maintain a maximum correlation, and any deviation that eventually occurs is due to the shading effects on PVMs. The deviation was verified when applying the shading induction experimental method, which, as can be seen in Figure 17 and Figure 18, oscillates around 1 A. Taking into account that the type of shading that does not activate the bypass diodes is the one that does not prevent the degradation phenomenon, the value of 1 A, established above, can be used as a threshold value that determines the formation of the PS to trigger the alert that the degrading causes, whose cumulative effect can produce irrecoverable degradation of the PVM, is active. In the experimental armature method carried out, for this type and model of PVMs, it was found that despite shading up to 18 solar cells, which formed two consecutive rows in series, the bypass diode did not trigger, and therefore the PVM safeguard system did not produce its effect, with which the degrading phenomenon of heating and dissipation of the power of the cells remains latent. The problem that occurs in practice is that not all forms of shading can cause the bypass diodes to trip, which would avoid the degrading effect; therefore, it must be the predictive diagnosis system that acts to automatically disconnect the affected panel if you want to stop the degrading effect.

A second experimental trial of partial shading cell was carried out to verify that if a partial shading occurs, although the reduction in the shaded area could in principle be associated with better dissipating conditions, that is not the case because the power dissipation increases with respect to that dissipated by a completely shaded cell (Figure 4). As the shaded area is reduced, the operating point of the shaded cell moves towards higher currents, so this causes an increase in energy dissipation. Therefore, regarding the phenomenon of PVM degradation, the conclusions support that partial shading of solar cells contributes more negatively than total shading [15].

In this work, as in other applications for predictive fault diagnosis, the aim is to apply a certain threshold value of comparative deviation from which we can determine that they are not small disturbances of little importance, but that a degradation shading effect is being produced. This effect produces unwanted heating on the active material of the affected solar cells that, if not avoided, will eventually cause the appearance of hot-spots on the PVM. Such degrading effects as hot-spots in the form of small blackish craters could be detected in an earlier phase in the PVMs used, which occupied the positions of SP1 and SP4, and which had to be replaced due to irrecoverable damage.

**Figure 19:**
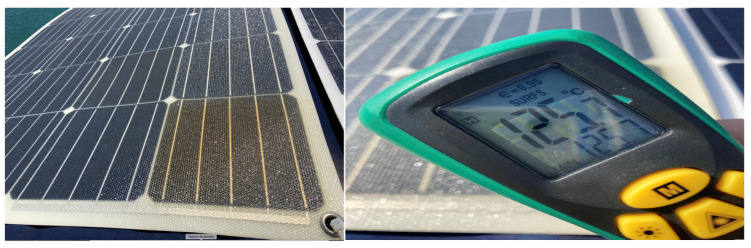
Brownish effect and temperature on a cell after a day of its installation.

The phenomenon of hot-spots, as commented previously, is commonly produced by shading effects, but not exclusively. It is also common to find it due to manufacturing defects in PVMs. During the experimental tests carried out, we had the opportunity to observe the spontaneous heating of a cell in one of the used panels, which was not due to an induced shading since the panel in question was not subjected at any time to the inductive shading method. It was a panel recently acquired, supplied, and installed two days before an irreversible anomaly of color change (brownish) was visually observed in a cell of one of the panels with respect to the color of its adjacent cells (Figure 19). Everything indicates that the cause of the failure was a manufacturing defect. At the initial moment of its visual detection, the heating of the cell reached temperatures of 125.7 °C. However, the decrease in the generation of current in this initial phase was not significant, which, from the point of the predictive diagnosis, turns out to be dangerous. In the affected solar cell, the temperature continued to increase until reaching 150 °C, after which, for safety reasons, the panel was disconnected.

**Figure 20:**
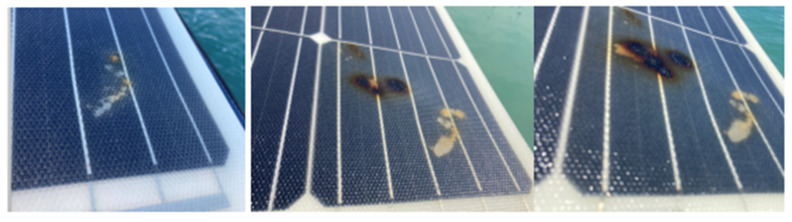
Development of hot-spots after three days of continuous use.

In the opposite part of the affected panel, the typical symptoms of the hot-spot began to appear rapidly with foci of whitish and brown discoloration, associated with greater point degradation because of the cell heating (Figure 20). Within three days, the location of the hot-spots spread, and the effect of the discoloration intensified with the emission of smoky threads that peeled off the base of the darker spots of the discoloration.

## 5. Conclusions

The proposed predictive diagnosis methodology for PVMs connected in a parallel system is effective in the sense that any shading process experienced in any panel is immediately detected as a reduction in the current generated in the PVM regulator. Therefore, the minimum threshold value obtained from the inductive experimental shading method can be used as a PS for the immediate detection and isolation of the shading event occurrence, as well as the automatic interruption of the degrading effect on the affected panel, avoiding the degradation process and therefore the appearance of a hot-spot.

The method is suitable for small photovoltaic installations that require the parallel connection of PVMs, such as isolated installations, vehicles and boats. Additionally, the proposed method can be generalized and appropriately scaled, if it is necessary to increase the number of PVMs of the photovoltaic installation. In that case, the variations introduced in the algorithm would only affect the size of the series data set, which will depend on the number N of PVMs, and as a consequence, N more data series are required to be incorporated into the comparative analysis. Since what underlies the diagnosis algorithm is the comparative analysis of the deviations of the quantitative values of the data series, at each sampling instant corresponding to each one of the panels, adding N more PVMs implies adding N additional data series in the data processing of each of the execution phases of the algorithm.

The predictive nature of the method lies in the fact that the diagnosis is not carried out in search of the event of an unrecoverable failure, but rather the diagnosis is focused on the early detection and isolation of an observable predictive symptom that, sufficiently in advance, triggers an alert indicating that a degradation process due to shadowing is taking place, whose cumulative effect of degradation, if not stopped, will cause the creation of an unrecoverable degradation state (failure).

It is found that in real-world applications where solar charge regulators are usually used, the number of possible parameters to be used as PS depends on the type of solar regulator used in the installation. In the case of the PWM regulator used in this initial study, the voltage value that can be observed is that of the battery to which it is connected. Therefore, it is not possible to observe what happens in the PVM voltage for the purposes of its possible use as a redundant electrical parameter in the appearance of shading phenomena.

In the case of using MPPT regulators, the energy that enters and leaves the regulator is the same as in PWM regulators, but the voltage and current are different from one side of the MPPT regulator to the other and, consequently, both electrical parameters are observable. In future works, the authors intend to use MPPT type regulators that will allow the observation of the specific voltage of the PVM individually.

## Figures and Tables

**Table 1:** PVI sensors and devices.

Device	Monitored Variables	Parameters	Range
DCM100	V, I	Battery Sense Voltage Range	0 to 50 VDC
		Battery Sense Voltage Accuracy	±100 mV
		Battery Current Range	0 to 200 A
		Battery Current Accuracy	±1%
TMP100 TRK3	Solar panel temperature	Measurement Range—Thermistor	(−20 °C to 80 °C)
DCR100		Maximum DC Switching Current	10 A
		DC Switching Voltage	<32 VDC
		Contact Resistance	<10 mΩ
		Current Sense Accuracy	±100 mA
USB100	Network PC connection	NMEA 2000^®^ Connector NMEA 2000^®^/USB Isolation	DeviceNet Micro-C Opto-Isolated
		USB Supported Signals	D+, D−, +5V, GND
		USB Auxiliary Power	+5 V < 50 mA
		USB Baud Rate	Up to 12 Mb/s
VDR100	Network data registration	USB Supported Signals	D+, D−, +5 V, GND
		USB Auxiliary Power	+5 V < 200 mA
		USB Baud Rate	Up to 12 Mb/s
		Ethernet Interface	100 Mb/s
PB200	Outside Temperature Wind speed and direction GPS	NMEA input A/+	
		NMEA output A/+	
		NMEA input B/−	
		NMEA output B/−	
		12 VDC+	
		12 VDC−/ground	
		Weather station instrument must be supplied with 12 VDC (±3 VDC) at 0.5 amp	
IMT DiRS485TC	Solar irradiance	Irradiance Measurement	up to 1500 W/m^2^
		Cell Temperature Measurement	−40 to +90 °C
		Working Temperature	−35 to 80 °C
		Voltage Supply	12 to 28 VDC
		Measuring Range	0 to 1500 W/m^2^
		Irradiance Protocol	MB: Modbus (RTU) MT: M&T protocol
PWM STECA 10 A	V, I	Service voltage	12 V o 24 V
		Permissible ambient temperature range	−10 °C y + 50 °C
		mA power consumption	12.5 mA
		Pulse width modulation (PWM) frequency	30 Hz

**Table 2:** PV Modules technical characteristics.

Peak Power (P_max_)	(W)	160 ^1^
Production Tolerance	(%)	±3
Maximum Power Current (I_mp_)	(A)	8.88
Maximum Power Voltage (V_mp_)	(V)	18.0
Short Circuit Current (I_sc_)	(A)	9.59
Open Circuit Voltage (V_oc_)	(V)	21.6
Weight	(Kg)	2.7
Dimensions	(mm)	670 × 1510 × 3
Maximum System Voltage	(VDC)	500
Cell Technology	Type	Monocrys
Cel Brand	Name	Ecosolar

^1^ All technical data at standard test conditions. AM = 1.5 E = 1000 W/m^2^ Tc = 25 °C.

**Table 3:** Predictive diagnosis algorithm quantitative values.

SP	Measured Current (A)														
SP1	5.6	5.9	6.4	6.8	7.3	7.4	7.8	8.1	7.5	7.3	6.9	6	5.8	5.1	4.2
SP2	6.7	7.1	7.6	8.1	8.4	8.4	8.7	8.8	8.2	7.8	7.4	6.3	6	5.35	4.4
SP3	6.4	6.7	6.9	7.2	7.4	7.2	7.2	7.2	6.4	5.7	5	4.1	3.5	2.4	0.8
SP4	7.25	7.5	7.6	7.7	7.3	7.5	7.4	7.2	6.2	5.5	4.70	3.6	2.9	2.2	1.5
MaxI_SP_t	7.25	7.5	7.6	8.1	8.4	8.4	8.7	8.8	8.2	7.8	7.4	6.3	6	5.35	4.4
DevI_SP1_t	1.65	1.6	1.2	1.3	1.1	1	0.9	0.7	0.7	0.5	0.5	0.3	0.2	0.25	0.2
DevI_SP2_t	0.55	0.4	0	0	0	0	0	0	0	0	0	0	0	0	0
DevI_SP3_t	0.85	0.8	0.7	0.9	1	1.2	1.5	1.6	1.8	2.1	2.4	2.2	2.5	2.95	3.6
DevI_SP4_t	0	0	0	0.4	1.1	0.9	1.3	1.6	2	2.3	2.7	2.7	3.1	3.15	2.9
DTV	SP1	SP1	SP1	SP1	SP1 SP3 SP4	SP1 SP3	SP3 SP4	SP3 SP4	SP3 SP4	SP3 SP4	SP3 SP4	SP3 SP4	SP3 SP4	SP3 SP4	SP3 SP4
Time	11:36	12:06	12:36	13:06	13:36	14:06	14:36	15:06	15:36	16:06	16:36	17:06	17:36	18:06	18:36

## Abbreviations

CPD	Current percent decrease
DC	Direct current
FD	Fault diagnosis
*I*	Current
*I_MPP_*	Maximum current in maximum power
IoT	Internet of things
*Isc*	Short circuit current
*I_SPi_*	Current generated by Solar Panel i
MTVPS	Minimum threshold value of the predictive symptom
NMEA	National Marine Electronics Association
NMEA 2000	NMEA Network
PFD	Predictive fault diagnosis
*P_MPP_*	Maximum power
PS	Predictor symptom
PVIs	Photovoltaic installations
PVMs	Photovoltaic modules or solar panels
PWM	Pulse-width modulation
SP	Solar panel
TCD	Total current decrease
*V*	Voltage
*V_DC_*	Direct current voltage
*V_MPP_*	Maximum voltage in maximum power
*Voc*	Open circuit voltage
